# A Systematic Review of Metabolic Syndrome: Key Correlated Pathologies and Non-Invasive Diagnostic Approaches

**DOI:** 10.3390/jcm13195880

**Published:** 2024-10-02

**Authors:** Francesco Giangregorio, Emilio Mosconi, Maria Grazia Debellis, Stella Provini, Ciro Esposito, Matteo Garolfi, Simona Oraka, Olga Kaloudi, Gunel Mustafazade, Raquel Marín-Baselga, Yale Tung-Chen

**Affiliations:** 1 Department of Internal Medicine, Codogno Hospital, Via Marconi 1, 26900 Codogno, Italy; francesco.giangregorio@asst-lodi.it (F.G.); emilio.mosconi@asst-lodi.it (E.M.); mariagrazia.debellis@asst-lodi.it (M.G.D.); stella.provini@ast-lodi.it (S.P.); ciro.esposito@asst-lodi.it (C.E.); matteo.garolfi@asst-lodi.it (M.G.); simona.oraka@gmail.com (S.O.); gunelmustafazade01@gmail.com (G.M.); 2 Department of Internal Medicine, Hospital Universitario La Paz, Paseo Castellana 241, 28046 Madrid, Spain; raquelzgz14@gmail.com

**Keywords:** metabolic syndrome, non-invasive assessment, liver fibrosis, cardiovascular disease, hepatic steatosis

## Abstract

**Background and Objectives:** Metabolic syndrome (MetS) is a condition marked by a complex array of physiological, biochemical, and metabolic abnormalities, including central obesity, insulin resistance, high blood pressure, and dyslipidemia (characterized by elevated triglycerides and reduced levels of high-density lipoproteins). The pathogenesis develops from the accumulation of lipid droplets in the hepatocyte (steatosis). This accumulation, in genetically predisposed subjects and with other external stimuli (intestinal dysbiosis, high caloric diet, physical inactivity, stress), activates the production of pro-inflammatory molecules, alter autophagy, and turn on the activity of hepatic stellate cells (HSCs), provoking the low grade chronic inflammation and the fibrosis. This syndrome is associated with a significantly increased risk of developing type 2 diabetes mellitus (T2D), cardiovascular diseases (CVD), vascular, renal, pneumologic, rheumatological, sexual, cutaneous syndromes and overall mortality, with the risk rising five- to seven-fold for T2DM, three-fold for CVD, and one and a half–fold for all-cause mortality. The purpose of this narrative review is to examine metabolic syndrome as a “systemic disease” and its interaction with major internal medicine conditions such as CVD, diabetes, renal failure, and respiratory failure. It is essential for internal medicine practitioners to approach this widespread condition in a “holistic” rather than a fragmented manner, particularly in Western countries. Additionally, it is important to be aware of the non-invasive tools available for assessing this condition. **Materials and Methods:** We conducted an exhaustive search on PubMed up to July 2024, focusing on terms related to metabolic syndrome and other pathologies (heart, Lung (COPD, asthma, pulmonary hypertension, OSAS) and kidney failure, vascular, rheumatological (osteoarthritis, rheumatoid arthritis), endocrinological, sexual pathologies and neoplastic risks. The review was managed in accordance with the PRISMA statement. Finally, we selected 300 studies (233 papers for the first search strategy and 67 for the second one). Our review included studies that provided insights into metabolic syndrome and non-invasive techniques for evaluating liver fibrosis and steatosis. Studies that were not conducted on humans, were published in languages other than English, or did not assess changes related to heart failure were excluded. **Results:** The findings revealed a clear correlation between metabolic syndrome and all the pathologies above described, indicating that non-invasive assessments of hepatic fibrosis and steatosis could potentially serve as markers for the severity and progression of the diseases. **Conclusions:** Metabolic syndrome is a multisystem disorder that impacts organs beyond the liver and disrupts the functioning of various organs. Notably, it is linked to a higher incidence of cardiovascular diseases, independent of traditional cardiovascular risk factors. Non-invasive assessments of hepatic fibrosis and fibrosis allow clinicians to evaluate cardiovascular risk. Additionally, the ability to assess liver steatosis may open new diagnostic, therapeutic, and prognostic avenues for managing metabolic syndrome and its complications, particularly cardiovascular disease, which is the leading cause of death in these patients.

## 1. Introduction

Nonalcoholic fatty liver disease (NAFLD) is highly prevalent across all continents, with a global prevalence rate of 24%. The highest rates are found in the Middle East (32%) and South America (31%), followed by Asia (27%), the USA (24%), and Europe (23%), while NAFLD is less common in Africa (14%) [1]. Other liver diseases, including viral hepatitis B and C, alcoholic liver disease, and hemochromatosis, also contribute significantly to the global prevalence of chronic liver disease [1].

A group of international experts recommended renaming NAFLD as “Metabolic Dysfunction-Associated Fatty Liver disease” (MAFLD) in 2020 [2]. Three years later [3], the term “Metabolic dysfunction-Associated Steatotic Liver Disease” (MASLD) was proposed, with diagnosis based on hepatic steatosis and one of five cardiovascular risk factors (1. BMI ≥ 25 kg/m^2^ OR WC > 94 cm (M) 80 cm, 2. Fasting serum glucose ≥5.6 mmol/L [100 mg/dl] OR 2-h post-load glucose levels ≥7.8 mmol/L [≥140 mg/dl] OR HbA1c ≥5.7% [39 mmol/L] OR type 2 diabetes OR treatment for type 2 diabetes, 3. Blood pressure ≥130/85 mmHg OR specific antihypertensive drug treatment, 4. Plasma triglycerides ≥1.70 mmol/L [150 mg/dl] OR lipid lowering treatment, 5. Plasma HDL-cholesterol ≤1.0 mmol/L [40 mg/dl] (M) and ≤1.3 mmol/L [50 mg/dl] (F) OR lipid lowering treatment), unlike MAFLD, which required two of seven metabolic dysfunction parameters. (1. Waist circumference ≥102/88 cm in Caucasian men and women, 2. Blood pressure ≥130/85 mmHg or specific drug treatment, 3. Plasma triglycerides ≥150 mg/dl (≥1.70 mmol/L) or specific drug treatment, 4. Plasma HDL-cholesterol <40 mg/dl (<1.0 mmol/L) for men and <50 mg/dl or specific drug treatment, 5. Prediabetes or HbA1c 5.7% to 6.4% [39 to 47 mmol/mol], 6. Homeostasis model assessment of insulin resistance score ≥2.5, 7. Plasma high-sensitivity C-reactive protein level >2 mg/L). Patients meeting both MASLD and alcohol-related fatty liver disease (ALD) criteria are classified as having MetALD [3].

The global pooled prevalence of NAFLD/MASLD is more relevant among patients with type 2 diabetes (T2D) and it corresponds to 65.33%. This prevalence rose from 55.86% in 1990–2004 to 68.81% in 2016–2021. The highest prevalence was seen in Eastern Europe (80.62%), followed by the Middle East (71.24%), and the lowest in Africa (53.10%). Among liver biopsy data, the global prevalence of NASH/MASH, significant fibrosis, and advanced fibrosis was 66.44%, 40.78%, and 15.49%, respectively. Pooled all-cause mortality was 16.79 per 1000 person-years (PY), with 4.19 for cardiac-specific, 6.10 for extrahepatic cancer-specific, and 2.15 for liver-specific mortality [4].

NAFLD, MALSD, and Metabolic Syndrome (MetS) share pathological processes of insulin resistance, chronic inflammation, and lipid dysregulation, which contribute to liver steatosis, fibrosis, and the development, not only of cardiovascular diseases (such as atherosclerosis, coronary artery disease, heart failure, and arrhythmias) [5] but also vascular (occlusive atherothrombotic disease of large- and medium-sized arteries) [6], pneumological [7], renal [8], metabolic (dislypidemia) [9], hyperuricemia [10], T2DM [11]), Sexual (male infertility [12,13] and Polycystic ovary syndrome [14]), cutaneous [15], neoplastic pathologies [16].

MASLD and increased mortality [17]. Therefore, it is crucial to have multidisciplinary care pathways to identify patients with MASLD who are at risk of liver and non-liver complications. These pathways should ensure that necessary lifestyle changes, management of metabolic risk factors, and potentially beneficial treatments are provided [17].

The initial clinical suspicion of metabolic syndrome often happens incidentally during an ultrasound performed for unrelated reasons, which reveals a “bright” liver. This finding is frequently considered incidental and not given much pathological significance. However, it often represents the “tip of the iceberg” of this silent yet dangerous condition, which is marked by a series of early metabolic abnormalities, including abnormal fat distribution, insulin resistance, atherogenic dyslipidemia, hypertension, and a proinflammatory and prothrombotic state [18]. These interconnected metabolic disorders have a significant impact on the cardiovascular system. In simple terms, inflammation increases insulin resistance, which leads to obesity while also exacerbating diabetes, high blood pressure, a prothrombotic state, and dyslipidemia. While inflammation and insulin resistance directly harm the cardiac muscle, the overall metabolic abnormalities contribute to cardiovascular complications [19,20].

The appearance of a bright liver echo pattern on an ultrasound is typically considered indicative of hepatic steatosis. Today, this ultrasound finding should prompt the consideration of metabolic syndrome in the patient. However, liver fibrosis can affect the sensitivity and specificity of the bright liver echo pattern, leading some to question its effectiveness as a diagnostic tool. Although the presence of liver fibrosis might reduce the accuracy of detecting a “bright liver” [21,22], the evidence supporting this concern is limited [22,23]. Nevertheless, the introduction of additional ultrasound features, such as posterior beam attenuation, has improved the sensitivity of ultrasound in detecting hepatic steatosis [21,24]. Previous research by Garra et al. [24] and Taylor et al. [25] showed that specific ultrasound parameters of steatosis are exclusively associated with fat accumulation in the liver cells. In 2006, Palmentieri et al. [26] concluded that the bright liver echo pattern indicates liver steatosis and that liver fibrosis does not interfere with ultrasound measurements. Posterior attenuation and/or skip areas are closely related to steatosis of 30% or more. Dasarathy et al. [27] found similar results: real-time ultrasound using a combination of sonographic findings has high specificity in diagnosing steatosis but tends to underestimate the prevalence of hepatic steatosis when fat content is below 20%. These findings were confirmed even in high-risk patients, such as those undergoing bariatric surgery [28]. In this patient group, Wu et al. [29] found that about three-quarters have liver steatosis, and about a quarter have fibrosis. One-third of patients with liver steatosis develop fibrosis without significant clinical symptoms. While ultrasound was only moderately effective in diagnosing liver steatosis, it was sufficient for clinical use in patients with a NAS score of 2 or higher and in those with obesity lasting more than 30 years. Recently, Ali et al. [30] demonstrated that the accuracy of Fibroscan in predicting significant liver fibrosis in morbidly obese patients is limited, with an accuracy of 71.3%. A model that combines hemoglobin A1c and alkaline phosphatase with liver stiffness measurements improves the accuracy of detecting significant fibrosis in this patient population. In conclusion, diagnosing, differentiating, and quantifying hepatic steatosis and fibrosis using conventional B-Mode ultrasound alone is challenging. However, it is essential for clinicians to understand metabolic syndrome and its interactions with major internal medicine conditions and to diagnose it non-invasively. The aim of this narrative review is to examine metabolic syndrome as a “systemic disease” and its interactions with key internal medicine conditions, such as cardiovascular diseases (hypertension, heart failure, arrhythmias, etc.), diabetes, renal failure, and respiratory failure. Clinicians must use this knowledge to address this widespread condition in a holistic and integrated manner, especially in Western countries. The aim of this review is to understand the knowledge of this systemic pathology, its related pathologies at the level of practically all body districts and, above all, the awareness that metabolic syndrome can be diagnosed, studied, and followed over time thanks to non-invasive, laboratory, and imaging methods.

## 2. Materials and Methods

### 2.1. Search Strategy

We carried out a thorough and systematic search on PubMed to identify pertinent literature, using a pre-established and reproducible search approach. The literature search was performed twice: the first for metabolic syndrome and its pathologies and the second for metabolic syndrome and its non-invasive assessment. For the first search, the terms included: (“Steatosis” OR “NASH” or “MAFLD” OR “MASLD” OR “Metabolic syndrome”) in combination with (“Heart Failure” [Mesh] OR “cardiac failure” OR “Cardiovascular Disease” OR “Kidney failure” OR “vascular Disease” OR “disease” OR “Diabetes”). For the second search, the terms included: (“Steatosis” OR “NASH” or “MASLD” OR “Metabolic syndrome”) in combination with “non invasive procedure” or “non invasive diagnosis” used for assessing hepatic steatosis (e.g., MRI, ultrasound), specific parameters measured (e.g., clinical systems like APRI, NFS, BARD, FIB-4 scores) 

### 2.2. Study Selection

The review was conducted in accordance with the PRISMA statement [31]. An electronic search of the Medline, EMBASE, and Cochrane Central Register of Controlled Trials (CENTRAL) databases was performed from inception until July 2024. The literature search was further extended by manually searching the reference lists of key articles. Two reviewers (FG and Y.T.-C.) assessed inclusion and exclusion criteria for each article independently. Any disagreements were resolved on a base of consensus.

We meticulously reviewed titles, abstracts, and full texts to assess their suitability according to predefined criteria. The inclusion criteria were as follows: (1) language: articles published in English; (2) study type: experimental, observational, and systematic reviews published as original research in peer-reviewed journals, limited to human studies; (3) population: adult patients diagnosed with any form of metabolic syndrome; (4) focus: studies exploring changes in the evaluation of metabolic syndrome and its effect on the progression or management of several correlated pathologies; (5) outcomes: studies that assessed the extent of fibrosis and steatosis and their association with metabolic diseases, especially heart failure. The exclusion criteria included: (1) case reports, opinion pieces, editorials, and studies available only in abstract form; (2) pediatric studies; (3) studies centered on populations without heart failure.

Two reviewers (F.G. and Y.TC.) assessed inclusion and exclusion criteria for each article independently. Any disagreements were resolved on a base of consensus. The following MeSH terms and keywords were used in the search: The literature search was extended by manual searching of reference lists of the essential articles. All references were exported with the use of EndNote X21™ and after eliminating duplicates, the identified study titles were screened. For the adequate ones, the abstracts were obtained, followed subsequently by a full-text examination for relevant content.

### 2.3. Data Extraction

After the initial screening, selected articles were subjected to a detailed review. Extracted data included: the first author, publication year, study location, sample size, and key findings related to hepatic steatosis assessment, as well as the management and outcomes of metabolic syndrome.

**Figure 1:**
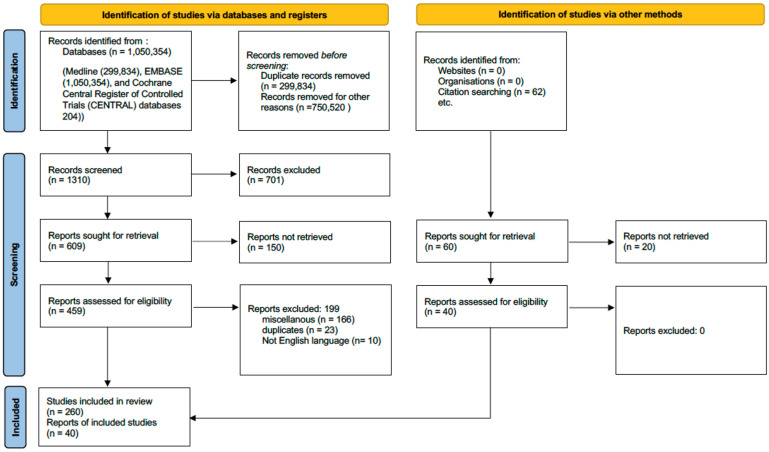
PRISMA 2020 flow diagram for metabolic syndrome and its pathologies and its non-invasive assessment.

Finally, we selected 300 studies (233 papers for the first search strategy and 67 for the second one) (Figure 1).

## 3. Results

### 3.1. Metabolic Syndrome: A Systemic Pathology

Metabolic syndrome is a condition marked by a complex set of physiological, biochemical, and metabolic factors, such as abdominal obesity, insulin resistance, high blood pressure, and dyslipidemia (elevated triglycerides and low levels of high-density lipoproteins) [32].

This syndrome significantly increases the risk of T2D by five to seven times, cardiovascular disease (CVD) by three times, and all-cause mortality by one and a half times [32]. Various international organizations and experts have proposed different criteria for defining MetS. The Modified National Cholesterol Education Program Adult Treatment Panel III (ATP III) [33] and the International Diabetes Federation (IDF) [18] are the most widely accepted. While both ATP III and IDF criteria include central obesity (measured by waist circumference with ethnicity- and gender-specific cutoffs), the IDF requires central obesity for a diagnosis, whereas ATP III considers it as one of several possible components.

### 3.2. From Steatosis to Metabolic Syndrome

Steatosis, commonly known as fatty liver, is triglyceride accumulation in hepatocytes, and a minimum excess overload of at least 5–10% [34], often associated with metabolic conditions like obesity and type 2 diabetes. It is a hallmark of non-alcoholic fatty liver disease (NAFLD) and can progress to more severe forms such as non-alcoholic steatohepatitis (NASH) if left unchecked. Until few years ago, liver biopsy was the only gold standard [35].

The development of steatosis is primarily driven by an imbalance between fat uptake, synthesis, and clearance in the liver. Excessive caloric intake, insulin resistance, and dysregulated lipid metabolism contribute to fat accumulation [35]. Over time, this buildup disrupts normal liver function [35].

Steatosis is often accompanied by low-grade, chronic inflammation [36]. In this state, inflammatory pathways are mildly activated over a prolonged period without the classical symptoms of acute inflammation. This inflammation stems from several factors including fat accumulation in liver cells, the release of pro-inflammatory cytokines from adipose tissue, and the activation of immune cells like macrophages [37].

The presence of steatosis triggers oxidative stress [38] and mitochondrial dysfunction [39], which lead to the production of reactive oxygen species (ROS) [38]. These, in turn, stimulate inflammatory pathways, causing a mild but sustained inflammatory response. This low-grade inflammation not only exacerbates liver damage but also contributes to insulin resistance and metabolic syndrome, creating a vicious cycle that accelerates disease progression [40].

A chronic inflammatory state contributes to metabolic syndrome, diabetes, and their cardiovascular consequences [41].

Autophagy is vital for maintaining cellular balance in response to internal stress, but its effectiveness declines with age and can be disrupted by overnutrition [42]. Autophagy disruption has been linked to metabolic diseases like obesity, T2D, and MASLD [42]. In T2D, autophagosome formation is increased in hyperglycemic or insulin-resistant patients, likely to clear damaged cell components. However, prolonged hyperglycemia and impaired insulin signaling may block autophagic flow, leading to the accumulation of faulty cellular parts, contributing to β-cell dysfunction [43]. In MASLD, evidence suggests autophagy inhibition as the disease progresses [43]. Yet, it is hard to determine whether autophagy is enhanced or suppressed in these diseases due to varying factors like tissue type and disease stage [43]. Recent studies suggest that using drugs to activate or restore autophagy can help improve liver function by clearing lipid droplets in liver cells, reducing the production of pro-inflammatory factors, and inhibiting hepatic stellate cells. This leads to an improvement in liver fibrosis and slows the progression of non-alcoholic fatty liver disease (NAFLD) [44].

In a healthy liver, quiescent hepatic stellate cells (HSCs) metabolize and store retinoids. When activated during liver fibrosis, these HSCs transform into myofibroblasts, lose their vitamin A, increase production of alpha-smooth muscle actin, and secrete proinflammatory mediators, collagens, and inhibitors of extracellular matrix (ECM) degradation. Activated HSCs are the key cells driving liver fibrosis. Additionally, the accumulation and activation of macrophages in response to hepatocyte death are crucial in triggering HSC activation and survival. The primary source of myofibroblasts is resident HSCs, which migrate to fibrotic areas to form scar tissue. While quiescent HSCs are uniform, activated HSCs/myofibroblasts show more variation. Inflammation arises from the response of various liver cells to hepatocyte death and injury-related signals, and this inflammation drives fibrosis by activating HSCs, which in turn, influence immune responses through cytokines and chemokines. Research also indicates that cellular stress responses play a role in fibrosis. Recent studies show that liver fibrosis can reverse even in advanced cirrhosis if the root cause is removed, which halts the activity of inflammatory and fibrogenic cells. However, despite many clinical trials for potential drug treatments, an approved antifibrotic therapy remains elusive [45].

### 3.3. Nafld/Masld and Nash/Mash

The term non-alcoholic steatohepatitis (NASH) was introduced in 1980 [46] to describe a condition resembling alcoholic hepatitis in patients without a history of alcohol abuse or infection from hepatotropic viruses, primarily in obese individuals [47,48]. Later, the term non-alcoholic fatty liver disease (NAFLD) was first used to describe a milder form of steatosis and eventually came to represent a wide range of liver conditions associated with fat accumulation, from simple fatty liver to cirrhosis [49]. From the beginning, insulin resistance was recognized as a key risk factor for NAFLD [50], and there was significant debate about whether NAFLD was a hepatic manifestation of metabolic syndrome [48]. In 1998, C.P. Day and O.F. James [51] introduced the “Two-Hit Hypothesis”, which identified insulin resistance as the primary trigger for MASLD development, while oxidative stress served as the second hit, contributing to the progression from simple steatosis to MASH [52]. Over time, the understanding of MASLMASLDD’s pathogenesis became more complex, leading to the “multiple hits hypothesis” [53] which suggests various factors, including diet [54], oxidative stress [55], gut microbiota [56], and genetics, may contribute to the onset and progression of NAFLD. Today, the definition of NAFLD encompasses various clinical phenotypes of non-alcoholic and non-viral liver fat accumulation, including metabolic NAFLD in obese patients and genetic NAFLD in lean individuals, with the latter often lacking insulin resistance [57,58]. A recent expert consensus [59] highlighted that NAFLD is still a diagnosis of exclusion, stressing the need for more precise diagnostic criteria that are better aligned with pathogenic processes [57,60,61]. This is aimed at creating a more uniform patient population for more effective drug research [59]. Additionally, experts recommend using biochemical scores (e.g., fatty liver index) for identifying fatty liver in large population studies. Fibrosis in MASLD can be categorized into stages F0 to F4, with F3 and F4 being considered advanced fibrosis [62]. Currently, patients with advanced fibrosis are advised to undergo hepatocellular carcinoma (HCC) surveillance based on a cost-benefit analysis [62]. Studies show a sharp increase in liver-related mortality in MAFLD patients with fibrosis stages greater than F2 compared to those with F0/1 [57]. The 2018 guidelines from the American Association for the Study of Liver Diseases (AASLD) [62] recommend using the NAFLD fibrosis score (NFS), the fibrosis-4 (FIB-4) index, vibration-controlled transient elastography (VCTE), and magnetic resonance elastography (MRE) for diagnosis. However, not all healthcare institutions have access to advanced imaging technologies like VCTE or MRE.

### 3.4. Risks Factors for Mets

The core metabolic features of MetS revolve around type 2 diabetes/insulin resistance [63,64,65,66], obesity (or abnormal fat distribution) [67,68,69], and hyperlipidemia [9,70,71], along with their related consequences: liver steatosis [17,72,73,74], cardiovascular alterations [5,75,76,77], and overall metabolic dysfunction [39,78]. This process primarily involves altered insulin function but also affects the actions of estradiol, testosterone, and glucocorticoids [79,80], along with dietary factors like excessive energy intake [81,82], imbalanced lipid or carbohydrate intake, and failure to maintain energy balance [83,84]. The nervous system initially regulates energy balance [81,85,86] by controlling appetite [87] and managing key hormones like insulin, glucocorticoids, testosterone, and 17β-estradiol [87]. Insulin plays a crucial role in this system, regulated by the pancreas, intestines, liver, and steroid hormones, primarily managing glucose availability and its storage or oxidation as energy [81,86].A defining feature of MetS is its strong connection to lifestyle factors common in modern societies [20]. MetS affects an increasing portion of the global population and is linked to common disorders driven by modern living conditions, such as abundant food supply, sedentary lifestyles, increased life expectancy, and strong social support systems. These conditions, while products of human societal progress, also present new challenges related to knowledge, behavior, and social organization, especially regarding food security. Despite the wide array of symptoms associated with MetS, a detailed analysis reveals that they largely stem from a common mechanism: chronic low-grade inflammation [88,89,90]. Today, many healthcare professionals and researchers consider MetS to be a condition of persistent, low-level inflammation [88,89,90].

### 3.5. A Summary of the Main Pathogenic Mechanisms of MetS Includes

(a) Hepatic steatosis, which impairs liver function, disrupts redox balance, and alters energy distribution. This is closely linked to lipid metabolism disorders, such as obesity and hyperlipidemia [84,91] affects related metabolic processes, like purine/urate metabolism [10,92].The liver plays a central role in processing nutrients, and damage here can lead to widespread dysfunction in other body systems. Liver steatosis, or non-alcoholic fatty liver disease (NAFLD), is often considered the primary metabolic disorder in MetS [11,93], as it disrupts energy management between the digestive system and circulation [94]. NAFLD is fundamentally tied to insulin dysfunction, which hampers the body’s ability to manage excess energy, leading to chronic liver inflammation and subsequent complications associated with MetS [95].

(b) Insulin resistance, glucose intolerance, and type 2 diabetes, are exacerbated by liver dysfunction, hormonal imbalances [96,97], and conditions stemming from ineffective insulin function [98], including issues with blood circulation and kidney function [99].

(c) Hormonal imbalances, including low levels of androgens and estrogens, which are linked to brain function [100] and insulin regulation [101].

(d) Corticosteroid dysfunction, which impacts hormone regulation, cellular growth, and immune responses [102]. Corticosteroids play a critical role in the defense system [103] by modulating and adjusting the immune response [104], both downplaying the immune response [105] but enhancing its effectivity [106]. Their function is necessary to prevent/correct the damages caused by inflammation [107]. Interrelated with all the previous points presented here (a–d) and the next one (e). An important aspect of corticosteroids is their regulation of blood vessel reactivity, affecting blood flow and pressure [108], largely in contraposition to DHEA (dehydroepiandrosterone) [109]. They also play a role in vascular reactivity, affecting blood pressure [69], and influencing behavior, and appetite regulation [110]. The relationship with neural transmission and the modulation of the complex nature of depression has been also acknowledged, but the mechanisms have not yet been fully established [111].

(e) Tissue and extracellular matrix disorders, which relate to energy distribution, fat deposition, and immune responses [112], and to the immediate relationships with ectopic fat deposition and obesity [113]. These disorders are connected to both obesity and immune system regulation [114]. Subcutaneous fat (SAT), which makes up about 80% of total adipose tissue, serves as the primary source of fatty acids for the liver and contributes to free fatty acids in circulating plasma [115]. SAT is linked to insulin resistance and visceral adipose tissue (VAT), with its quantity correlating positively with metabolic syndrome factor scores, regardless of age and sex [116]. Inflammatory cell infiltration, including macrophages and inflammatory cytokines, plays a key role in adipose tissue inflammation. Obese individuals often exhibit an inflamed adipose phenotype characterized by macrophage accumulation in “crown-like structures”, leading to impaired local vasodilation and increased insulin resistance. The presence of macrophages, marked by CD68 expression, is associated with elevated plasma levels of high-sensitivity C-reactive protein (hsCRP) and local TNFα concentrations. Adipokines [117] are suggested to be significant endocrine mediators influencing adipose tissue function. The following proteins are classified as adipokines: (1) Leptin [118], which indicates fat body mass; (2) Adiponectin [119], a marker of insulin sensitivity and adipose function; (3) Ghrelin [118], which stimulates appetite and visceral fat accumulation; (4) Vaspin [120], which improves glucose metabolism and reduces food intake; (5) Retinol binding protein 4 [121], a marker of insulin sensitivity and fat distribution; (6) Adropin and Apelin [122], which improves glucose metabolism; (7) Progranulin and FAM19A5 [123,124], markers of macrophage infiltration in adipose tissue; (8) Omentin [120], linked to visceral fat mass; (9) Resistin and Chemerin [125], associated with inflammation and insulin resistance; and (10) Fetuin [126], which reflects liver fat content.

In early-stage METS, plasma adipokines contribute to systemic inflammation [116]. Leptin, adiponectin, and resistin are key markers, along with pro-inflammatory cytokines like TNFα, IL-1β, and IL-6, which also play roles in insulin resistance [127]. Decreasing TNFα may reduce airway inflammation and eosinophil recruitment, potentially influencing asthma in obese patients, who might respond better to leukotriene modifiers than to inhaled corticosteroids during asthma exacerbations [128].

(f) Microbiota interactions and immune responses to external agents, leading to conditions like asthma, allergies, and skin disorders. These reactions, while localized, can have significant systemic effects, particularly when they involve overactive immune responses [129].

(g) Potential links to rheumatic diseases, such as osteoarthritis and psoriasis, which may be tied to MetS through shared mechanisms involving immune regulation and tissue homeostasis [130,131],

Metabolic syndrome pathophysiology demonstrates that a relationship among MetS, diabetes, Chronic Kidney failure Disease (CKD), atherosclerotic cardiovascular disease (ASCVD) and HF is bidirectional: Nichols et al. recently demonstrated, in a large population, a bidirectional association among Type 2 Diabetes, Chronic Kidney Failure and atherosclerotic cardiovascular disease (ASCVD) [132]: the authors concluded that the presence of T2DM, CKD, HF, and ASCVD each conveys risk on the others. A cardiometabolic renal syndrome comprising these conditions may be an important disease entity that requires a comprehensive treatment approach. A recent paper [133] studied a population of patients aged ≥18 years with new-onset T2DM, without renal disease or HF at baseline and incidence of CKD was higher than that of HF: 17.6 vs. 10.6 person-years, respectively, but incident HF was associated with a higher adjusted mortality than incident CKD. The presence of either condition (vs. CKD/HF-free status) was associated with a three-fold hazard of death, whereas concomitant HF and CKD conferred a six to seven-fold adjusted hazard of mortality.

### 3.6. Metabolic Syndrome: A Systemic Disease

The vascular damage caused by Metabolic Syndrome affects the functioning of various organs, including the kidneys [134,135], retina [136,137], adipose tissue [136,138], and lungs [139,140]. Pulmonary function is severely impacted by MetS [117,141,142], which is consistent with broader cardiovascular complications [143]. Inflammation in the lungs likely exacerbates the chronicity and severity of diseases caused by infection, toxicity, autoimmunity, or blockages, resulting in fibrosis and irreversible loss of function [144], such as in asthma and chronic obstructive pulmonary disease (COPD) disease [145,146]. MetS also increases lung vulnerability to infections [147,148] and cancer [16].

### 3.7. Steatosis, Mash and Metabolic Syndrome and Heart Failure

#### 3.7.1. Steatosis and Heart Failure

Clinically, there is a clear connection between liver steatosis and heart failure. Park et al. [149] explored the relationship between liver steatosis and/or fibrosis and the onset of incident heart failure (iHF), hospitalized heart failure (hHF), mortality, and cardiovascular (CV) death in both the general population and in heart failure patients. Their findings showed that 28,524 individuals (3.7%) in the general population and 1422 (19.1%) patients with pre-existing heart failure developed iHF and hHF, respectively. In a model adjusted for multiple variables, participants with a fatty liver index (FLI) of 60 or higher had an increased risk for iHF (hazard ratio [HR], 1.30, 95% confidence interval [CI], 1.24–1.36), hHF (HR 1.54, 95% CI 1.44–1.66), all-cause mortality (HR 1.62, 95% CI 1.54–1.70), and CV mortality (HR 1.41, 95% CI 1.22–1.63) in the general population, as well as an increased risk for hHF (HR 1.26, 95% CI 1.21–1.54) and all-cause mortality (HR 1.54, 95% CI 1.24–1.92) in heart failure patients, compared to those with an FLI below 20. Among participants with non-alcoholic fatty liver disease (NAFLD), advanced liver fibrosis was linked to a higher risk for iHF, hHF, and all-cause mortality in the general population, as well as increased all-cause and CV mortality in heart failure patients (all *p* < 0.05). In a follow-up study in 2022, the same researchers [150] found that persistent liver steatosis raised the risk of iHF, hHF, and mortality, including CV- and liver-related deaths, compared to those without a history of steatosis (all *p* < 0.05). New-onset hepatic steatosis was also associated with an increased risk of iHF and mortality, including CV- and liver-related deaths (all *p* < 0.05). In contrast, regression of hepatic steatosis, compared to persistent steatosis, was linked to a lower risk of iHF, hHF, and liver-related mortality (iHF, HR [95% CI] 0.800 [0.691–0.925]; hHF, 0.645 [0.514–0.810]; liver-related mortality, 0.434 [0.223–0.846]).

#### 3.7.2. Mash and Heart Failure

Insulin resistance (IR) is an independent predictor of Mets and heart failure (HF), regardless of the presence of diabetes or other known HF risk factors [151]. Several mechanisms could explain this association. First, the formation of advanced glycation end products (AGEs) activates the renin-angiotensin-aldosterone system, leading to increased myocardial collagen crosslinking and stiffness. Additionally, insulin functions as a myocardial growth factor, and hyperinsulinemia has been shown in animal studies to cause myocardial hypertrophy, reduce cardiac output, and increase sodium retention [152]. This sodium retention may result in decompensation in individuals with subclinical myocardial dysfunction due to volume overload [153]. Hyperinsulinemia also stimulates the sympathetic nervous system, which negatively affects cardiac structure and function, leading to impaired cardiac innervation and potentially contributing to HF [154]. Moreover, IR is associated with heightened sensitivity to angiotensin II [155], which promotes cellular hypertrophy and collagen production, ultimately leading to myocardial hypertrophy, fibrosis, and potential HF. It is important to note that HF itself can induce a state of insulin resistance [156]. In HF, reduced arterial blood volume triggers cardiovascular baroreceptors, which activate the sympathetic nervous system, causing the release of norepinephrine [157]. This response enhances myocardial contractility and increases peripheral vascular resistance through the activation of alpha and beta-adrenergic receptors [158]. Elevated norepinephrine levels, in turn, impair insulin sensitivity and glucose tolerance [159]. In addition to sympathetic overactivity, inflammation, the side effects of HF medications (such as beta-blockers and angiotensin-converting enzyme inhibitors) and decreased physical activity may also contribute to the development of IR in non-diabetic HF patients. Animal studies [160] have shown that HF impairs glucose uptake, likely due to reduced translocation of the glucose transporter GLUT4 to the cell membrane [161]. The resulting intracellular glucose deficiency forces cardiomyocytes to rely on free fatty acids, which leads to a less efficient metabolic state, ultimately worsening HF progression [162]. These preclinical findings suggest that HF patients without diabetes are at an increased risk of developing IR. Furthermore, IR is strongly linked to poorer survival outcomes in HF patients [163].

#### 3.7.3. Metabolic Syndrome and Heart Failure

Individuals with MetS are twice as likely to develop heart failure (HF) compared to the general population [164,165], with 22% to 68% of HF patients showing characteristics of MetS [75,166,167]. Evidence strongly supports the view that MetS is a significant risk factor for developing HF. The key link between MetS and HF is insulin resistance (IR) [168], which is a predictor of HF, independent of diabetes and other established risk factors. In fact, patients with HF but without diabetes are more likely to develop IR, and this condition is closely related to survival rates in HF patients [151].

Heart failure is a major public health concern, affecting 3% of the general population and being the leading cause of mortality, hospitalizations, and healthcare costs in individuals over 65 [162,169]. The lifetime cost of managing HF is approximately USD 109,541 per person [170], with over 75% of this cost attributed to hospitalizations, especially in the final months of life [171]. Cardiovascular diseases are common in individuals with MetS and represent its most frequent outcomes. These include increased risks of hypertension, atherosclerosis, stroke, arrhythmias, and heart failure [172], which may arise due to coronary insufficiency, loss of heart function due to tissue damage or rhythm disturbances, or the degenerative processes affecting heart function [173]. The root of these cardiovascular issues lies in distorted metabolic regulation, potentially leading to sarcopenia, tissue damage, altered blood vessel function, and issues with heartbeat rhythm or blood coagulation.

Cardiovascular diseases are a leading cause of death and disability associated with MetS, although the total risk is still lower than the risk posed by these conditions when considered individually. A substantial body of research shows that HF patients face numerous metabolic comorbidities, significantly affecting their prognosis. Cardiovascular diseases are almost omnipresent in populations with MetS, and constitute, perhaps, its most common consequences [174]. They can be summarized by an increased risk of Hypertension [175,176], atherosclerosis [177], stroke [178], arrhythmias [179], and heart failure [180]: coronary insufficiency or claudication [19], loss of heart function because of damaged tissue or contractile rhythm, signal transmission, or bringing up altered function because of degenerative processes [181]. The origin of these cardiovascular pathologies is a consequence of distorted metabolic function and regulation, which may result in sarcopenia [182], damaged tissue [183], altered vessel function [184], loss of heartbeat rhythm or dysfunctional signal transmission [185], altered blood coagulation [186].

Cardiovascular diseases are a main cause of death and incapacity, attributable to MetS [25], but its total risk remains lower than those of these individual disorders taken independently [26]. A consistent body of evidence demonstrated that patients with HF are burdened by several metabolic comorbidities that dramatically impact on outcomes [187].

### 3.8. Paradox Effect: The “Reverse Epidemiology”

Several studies have shown that in patients hospitalized with a confirmed diagnosis of heart failure (HF), certain established cardiovascular risk factors, such as obesity, hypertension, and hypercholesterolemia, appear to have a protective effect on clinical outcomes. This phenomenon is referred to as “reverse epidemiology” [188,189,190]. Key studies on chronic HF have revealed that BMI is inversely related to long-term mortality: patients with a higher BMI exhibit lower mortality rates compared to those with a lower BMI, regardless of left ventricular ejection fraction [189,191]. This phenomenon, known as the “obesity paradox”, has not been fully explained. One possible reason is that a higher BMI may offer better protection against the malnutrition-inflammation syndrome often seen in chronic HF and the cachexia associated with advanced HF. A similar paradox is observed in the relationship between HF and hypertension. In advanced stages of HF, higher blood pressure is linked to better outcomes, as shown in sub-analyses from the Digitalis Investigation Group trial database [192] and a meta-analysis of 10 studies [193]. These studies found that a 10 mm Hg increase in systolic blood pressure was associated with a 13% reduction in mortality among the HF population. The HF-hypertension relationship varies based on the severity of HF: in milder cases, the relationship follows a U-shape, while in more severe cases, it is linear [194]. There is strong evidence that type 2 diabetes in patients with HF is linked to increased morbidity and mortality, with an additional risk of 1.3 to 2.0, particularly in those with an ischemic cause. However, the effect of glycemic control, as measured by hemoglobin A1c levels, on HF outcomes is less clear. Some observational studies have shown a U-shaped or inverse relationship between HbA1c and mortality in HF patients with T2DM [195]. Most research indicates that patients with strict glycemic control (HbA1c < 7%) have worse outcomes than those with more moderate control (HbA1c > 7%). Nevertheless, the lack of clinical trials specifically targeting glucose management in HF patients makes it difficult to establish clear glucose control guidelines for this population [196]. In patients with established HF, several analyses have demonstrated an inverse correlation between cholesterol levels and outcomes, with lower cholesterol levels independently associated with higher mortality and higher cholesterol levels linked to better survival [188].

### 3.9. Metabolic Syndrome and Vascular Pathologies

Several studies have explored the relationship between NAFLD and non-cardiac atherosclerotic cardiovascular disease (ASCVD). One study [197] found NAFLD was linked to a lower stroke risk, with no racial differences but a significant sex-related interaction. Another study [198] showed no significant association between NAFLD and stroke incidence. However, a Korean cohort study [199] found that patients with NAFLD and liver fibrosis had a higher risk of carotid atherosclerosis progression. A cross-sectional study indicated that elevated liver enzymes were associated with a higher risk of stroke [200]. A meta-analysis [201] found NAFLD increased the risk of ischemic stroke, with NASH [202] being a stronger predictor than simple steatosis. Additional studies found correlations between NAFLD and carotid intima-media thickness (cIMT) [203], peripheral arterial disease (PAD) [204], and arterial stiffness [205]. Advanced liver fibrosis, hypertension, and older age were identified as independent risk factors for arterial stiffness in NAFLD patients [206]. NAFLD/NASH was also linked to abdominal aortic aneurysm [207], even after adjusting for other factors like BMI and smoking.

### 3.10. Metabolic Syndrome and Kidney Failure

The global prevalence of kidney disease is rising, driven by increasing rates of obesity, diabetes, and metabolic syndrome. These conditions, along with chronic kidney disease (CKD), create a heavy financial burden on healthcare systems. Despite treatments like RAAS inhibitors, blood pressure, and glycemic control, newer drugs such as SGLT-2 inhibitors and GLP-1 receptor agonists, high-risk obese and diabetic populations remain vulnerable to progressing to end-stage renal disease (ESRD). MetS leads to endothelial dysfunction and a hypercoagulable state, which particularly affects the kidney due to its vascular structure. MetS accelerates the onset and progression of CKD [208]. Cardiometabolic conditions, particularly type 2 diabetes and heart failure (HF), are significant independent risk factors for the onset and progression of chronic kidney disease (CKD). Additionally, CKD and its progression are independently linked to the development of T2DM, atherosclerotic cardiovascular disease (ASCVD), and HF. The coexistence of these conditions further heightens the risk of CKD onset or progression. This bidirectional relationship between cardiometabolic and renal diseases suggests a broader concept of cardiorenal syndrome, which includes ASCVD, T2DM, CKD, and HF, forming a cardio-renal-metabolic disease continuum [132].

A study [209] including 186 diabetics and 57 controls found that 66.6% of T2D patients had non-alcoholic fatty liver disease (NAFLD). T2D patients with NAFLD had a greater metabolic burden and higher median liver stiffness compared to non-NAFLD diabetics. Over a median follow-up of 5.6 years, 17.7% of T2D patients developed adverse outcomes (OCE) compared to 7% of controls. There was no significant difference in overall clinical events rates between NAFLD and non-NAFLD diabetics. NAFLD was linked to chronic kidney disease (CKD), while T2D complications and subclinical cardiovascular disease rates were similar. Higher liver stiffness, older age, and male gender were independently associated with adverse outcomes in T2D. The study concludes that liver evaluation is recommended for high-risk T2D patients for early referral to specialized care.

### 3.11. Metabolic Syndrome and Respiratory Failure

Asthma: a meta-analysis [210] showed that the risk for asthma in overweight and obese men and women was similar, Patients with high body mass index (BMI) have an increased risk of clinically relevant asthma and respond less to corticosteroid treatment [210].

Leptin, adiponectin, and ghrelin are thought to impact asthma development and control, regardless of obesity or gender [211]. Obese individuals generally experience poorer asthma control and respond less effectively to treatment [212]. Gastric bypass surgery may help improve asthma outcomes in obese patients [210]. Additionally, central adiposity triggers adipokine expression, leading to sympathetic nervous system overactivity, contributing to hypertension and metabolic issues, and possibly affecting eosinophil recruitment in obese children [213].

Dyslipidemia alters immune cell trafficking to the lungs, playing a role in lung conditions such as acute lung injury, asthma, and pneumonia [213]. Cholesterol levels impact lung physiology, with LDL impairing surfactant function, while HDL supports lung health by delivering antioxidants and promoting surfactant production [213].

Chronic Obstructive Pulmonary Disease (COPD): According to the International Diabetes Federation (IDF) [18], while smoking and COPD are not canonical risk factors for metabolic syndrome, recent clinical findings suggest a strong association between the two [214,215]. MetS is found to be twice as common in COPD patients compared to the general population, with a prevalence ranging from 21% to 62% [216]. Almost 50% of COPD patients exhibit one or more components of MetS, which significantly increases the risk of type 2 diabetes mellitus, cardiovascular disease (CVD), and stroke [217]. The coexistence of COPD and MetS leads to more severe disease manifestations, including increased dyspnea and greater medication requirements. MetS is present in younger COPD patients and those with milder forms of the disease [216]. Increased insulin resistance in COPD patients with MetS may contribute to the development of T2DM. Although the exact cause of MetS in COPD is not well understood, it is thought to be multifactorial, with common contributing factors including oxidative stress, inflammatory cytokines, and physical inactivity Additionally, adipose tissue inflammation is recognized as an important mechanism linking COPD and MetS [217]. Increased oxidative stress and the “spillover” of lung inflammation into systemic circulation are crucial in linking COPD to its comorbidities, including MetS [214,216]. Elevated inflammatory biomarkers and cytokines in the blood are associated with low-grade systemic inflammation, a key mediator of MetS, promoting insulin resistance and atherosclerotic plaque formation.

On the opposite, the development of MetS in patients with COPD is influenced by repeated smoking and exposure to harmful particles like occupational dust and air pollution [217]. These exposures trigger an inflammatory response, leading to increased infiltration of macrophages and neutrophils in the lungs. The combination of inhaled oxidants and endogenous oxidative stress results in oxidative damage to various cellular components, activating lung resident cells and perpetuating inflammation [216]. This creates a cycle of chronic inflammation and oxidative stress that disrupts the balance of proteases and anti-proteases, impairs tissue repair, accelerates apoptosis, and causes tissue destruction. Consequently, pro-inflammatory mediators spill into the systemic circulation, promoting cardiovascular disease and metabolic abnormalities, which are interconnected with other comorbidities [216]. Additionally, impaired lung function reduces oxygen exchange efficiency, leading to hypoxemia, which, along with physical inactivity, contributes to the progression of MetS and other comorbid conditions [216].

Obstructive sleep apnea syndrome (OSAS): is a prevalent condition characterized by repeated episodes of halted airflow (apneas), resulting in decreased oxygen levels in the blood and disruptions in sleep [218]. This syndrome can lead to several consequences that significantly impact an individual’s quality of life, including excessive daytime drowsiness, cognitive decline, and various endocrine and metabolic issues [219]. Metabolic syndrome (MS), which is also becoming a widespread public health concern, may be associated with OSAS. Specifically, having OSAS may elevate the risk of developing certain components of metabolic syndrome. Furthermore, clinical research [220] has demonstrated a statistically significant relationship between the severity of OSAS and conditions such as obesity, hypertension, diabetes mellitus, dyslipidemia, and metabolic syndrome.

Pulmonary Hypertension: Pulmonary hypertension (PH) is a condition with high morbidity that can result from various causes, such as lung tissue damage with hypoxia, elevated pressure in the left atrium, chronic blood clots in the lungs, or primary pulmonary vascular disorders, known as pulmonary arterial hypertension (PAH) [221]. Growing basic and clinical research suggests that PH and PAH may be better understood as syndromes. In fact, neurohormonal and metabolic imbalances may contribute to the development and progression of both conditions [222]. A study [223] aimed to determine the prevalence of metabolic syndrome (MetS) in patients with pulmonary hypertension (PH), including various diagnostic groups such as pulmonary arterial hypertension (PAH), PH associated with lung disease, and other subgroups. The researchers conducted a retrospective review of patient records from the Mayo Clinic between 1990 and 2013, including 640 patients confirmed to have PH via right heart catheterization. MetS was defined by the presence of at least three conditions: hypertension, diabetes, hyperlipidemia, or a BMI over 30. Results showed that 39% of PH patients had MetS, with the highest prevalence in group 2 (PH due to left heart disease) and group 3 (PH associated with lung disease). After adjusting for age, sex, and race, groups 2 and 3 had significantly higher odds of MetS compared to group 1 (PAH). The conclusion highlights that MetS is more common in patients with PH related to left heart or lung disease.

### 3.12. Metabolic Syndrome and Arthritis

Patients with arthritic diseases, such as rheumatoid arthritis and osteoarthritis, have a higher risk of cardiovascular disease (CVD). Obesity-related chronic inflammation contributes significantly to the rise in MetS, and adipokines, which regulate metabolism, also play a role in autoimmune and inflammatory processes in arthritis. Thus, MetS and the dysregulated secretion of pro-inflammatory adipokines like leptin, adiponectin, and lipocalin-2 affect the immune system, MetS, and arthritis [115,116].

Osteoarthritis [224], the most prevalent type of arthritis, is a progressive, degenerative, and multifaceted disease that impacts the entire joint, including the articular cartilage, meniscus, ligaments, bone, and synovium. It is marked by molecular, anatomical, and physiological disruptions, such as abnormal joint tissue metabolism, cartilage breakdown, bone remodeling, osteophyte development, inflammation, and the loss of normal joint function.

It has been confirmed that the occurrence of metabolic syndrome (MetS) is more common in individuals with osteoarthritis (OA) [225]. Factors such as inflammation, oxidative stress, shared metabolites, and endothelial dysfunction have been suggested as potential mechanisms connecting the development of metabolic OA to MetS.

Rheumatoid arthritis (RA) is a chronic inflammatory joint disease characterized by swelling of the synovial membrane, destruction of cartilage and bone, the production of autoantibodies (such as rheumatoid factor and anti-citrullinated protein antibody), and various systemic complications including pulmonary, skeletal, cardiovascular, and psychological issues [226]. Studies have shown that metabolic syndrome (MetS) is commonly found in RA patients, and those with MetS have a higher risk of developing moderate-to-severe RA compared to those without it [227].

### 3.13. Metabolic Syndrome and Other Diseases

MetS has also been linked to various sexual, skin, and neoplastic pathologies.

Sexual Health: MetS is associated with sexual dysfunction in both men and women. In men, it can lead to erectile dysfunction due to impaired blood flow and hormonal imbalances, particularly low testosterone levels [12,13]. Women may experience reduced libido and arousal difficulties, often influenced by hormonal changes related to obesity and insulin resistance. MetS is also correlated with Polycystic ovary syndrome [14].

Skin Conditions: Individuals with MetS are at a higher risk for several skin disorders. Conditions like acanthosis nigricans [228], characterized by dark, velvety patches of skin, often signal insulin resistance. Additionally, skin infections and inflammatory conditions, such as psoriasis [229], are more prevalent, potentially due to systemic inflammation and altered immune responses linked to MetS.

Neoplastic Diseases: MetS is a significant risk factor for various cancers [230], including breast, colorectal, and endometrial cancers. The underlying mechanisms may involve chronic inflammation, hormonal changes, and insulin resistance, which can promote tumor growth and development. The association between obesity and cancer is particularly strong, as excess adipose tissue contributes to a pro-inflammatory state that may facilitate cancer progression.

### 3.14. Non Invasive Methods for Hepatic Fibrosis and Steatosis

Knowledge of METS allows us to identify it better and earlier and to search for it in many types of patients; furthermore, clinicians can study and follow up this “Systemic” pathology thanks to the use of non-invasive methods, both clinical-laboratory and imaging. For this reason, it is useful to know these methods for hepatic FIBROSIS and STEATOSIS evaluation.

## 4. Overview of Non-Invasive Methods for Hepatic Fibrosis

Liver biopsy was the most reliable approach for identifying the presence of steatohepatitis and fibrosis in patients with NAFLD, but it is generally acknowledged that biopsy is limited by cost, sampling error, and procedure-related morbidity and mortality [231]. The importance of liver biopsy in metabolic syndrome could be reevaluated in light of the new emerging role of genetics in precision medicine [232]. Anyway, many authors have always tried to develop non-invasive systems, based on anthropomorphic, clinical and laboratory data, to calculate and predict the presence of fibrosis in liver patients. The main scores (which will be discussed briefly in this review) are summarized in Table 1.

The non-invasive study of hepatic fibrosis (Table 1) and steatosis (Table 2) scores were developed in the hepatology field; therefore, they have value not only in the hepatological but also in the cardiovascular and metabolic fields and could open the doors to future diagnostic flow charts in the diagnosis and therapy of these pathologies.

### 4.1. APRI

In 2003, the AST to platelet ratio index (APRI)) [233] was introduced to highlight the contrasting effects of liver fibrosis on AST levels and platelet counts in patients with HCV-related hepatitis. The Area Under the Curve (AUC) for APRI was 0.80 for predicting significant fibrosis and 0.89 for cirrhosis in the training group. By using optimized cut-off values, significant fibrosis could be accurately predicted in 51% of cases and cirrhosis in 81% of cases. A meta-analysis [240] later demonstrated that in HBV-related patients, both APRI and FIB-4 showed moderate accuracy for detecting significant fibrosis, advanced fibrosis, and cirrhosis. The mean Area Under the Summary Receiver Operating Characteristic (AUSROC) for FIB-4 was higher than that for APRI (0.76 vs. 0.72) in predicting significant fibrosis. When analyzed together, FIB-4 had an AUC of 0.7844 (95% CI: 0.7450–0.8238), while APRI had a value of 0.7407. A further review [241] focused on HCV-related patients compared the effectiveness of transient elastography to the AST to platelet ratio index in staging liver fibrosis in chronic hepatitis C patients. The findings showed that for predicting significant fibrosis, transient elastography and APRI had diagnostic odds ratios of 11.70 (95% CI: 7.13–19.21) and 8.56 (95% CI: 4.90–14.94) respectively. For predicting cirrhosis, transient elastography had a diagnostic odds ratio of 66.49 (95% CI: 23.71–186.48) compared to APRI’s 7.47 (95% CI: 4.88–11.43). The authors concluded that there was no significant evidence proving transient elastography’s superiority over APRI in predicting significant fibrosis, but it was more effective in predicting cirrhosis.

### 4.2. NFS

In a large group of individuals with nonalcoholic fatty liver disease (NAFLD) and advanced liver fibrosis, a new non-invasive scoring system was developed to assess six independent indicators of advanced fibrosis. These indicators include age, hyperglycemia, body mass index (BMI), platelet count, albumin levels, and the AST/ALT ratio. This system, known as the NAFLD fibrosis score (NFS) [234], is calculated using the following formula: NAFLD fibrosis score = −1.675 + 0.037 × age (years) + 0.094 × BMI (kg/m^2^) + 1.13 × IFG/diabetes (1 if yes, 0 if no) + 0.99 × AST/ALT ratio—0.013 × platelet count (×10^9^/L) − 0.66 × albumin (g/dL). By applying a low cutoff score of −1.455, advanced fibrosis could be excluded with high accuracy, showing a negative predictive value of 93% in the estimation group and 88% in the validation group. On the other hand, using a high cutoff score of 0.676 allowed for the accurate diagnosis of advanced fibrosis, with a positive predictive value of 90% in the estimation group and 82% in the validation group.

### 4.3. BARD SCORE

The BARD score [235], which combines BMI > 28, an AST/ALT ratio > 0.8, and the presence of diabetes, was developed to enhance the clinical utility of easily accessible parameters in predicting MASH fibrosis. The score showed a composite measure of sensitivity and specificity (AUC) through ROC analysis, with AUC values ranging from 0.81 to 0.83 across various validation subsets. The overall AUC for predicting advanced fibrosis in the total sample was 0.81. In this study, the BARD score was also compared to the NAFLD fibrosis score in a group of 92 patients. The AUC for the NAFLD fibrosis score was 0.74, with a positive predictive value (PPV) of 26% and a negative predictive value (NPV) of 91%. This comparison indicates that the BARD score, which is simpler to calculate, is at least as effective as the more complex NAFLD fibrosis score in ruling out advanced fibrosis in patients.

### 4.4. FIB-4

The FIB-4 index is a scoring system based on routine blood tests (age, AST, ALT, and platelet count), originally developed to assess liver fibrosis in patients co-infected with HIV and HCV [236]. Initial regression analysis identified four variables as independent predictors of fibrosis: age, AST, PT-INR, and platelet count in a study involving 505 HIV/HCV co-infected patients. In a second model with 553 patients, ALT replaced PT-INR, focusing on age, AST, platelet count, and ALT [236]. The FIB-4 index has proven effective in identifying severe fibrosis (F3/4) in HCV mono-infected patients [237]. For patients who have cleared HCV but have FIB-4 scores ≥ 3.25, the risk remains high enough to justify HCC (liver cancer) surveillance [242]. In patients with chronic HBV infection who do not have cirrhosis, a low FIB-4 score is useful for predicting minimal risk of liver-related events, such as cancer, progression to cirrhosis, and mortality. Thus, the FIB-4 index has become a non-invasive test (NIT) for detecting severe fibrosis or assessing the risk of liver-related events in patients with chronic viral hepatitis [243,244,245]. In metabolic-associated fatty liver disease (MAFLD), a study by Shah and colleagues [246], involving 541 MAFLD patients, found that the FIB-4 index had superior diagnostic accuracy compared to other serum markers for assessing liver fibrosis. A meta-analysis [247], comparing the FIB-4 index, NAFLD fibrosis score (NFS), and BARD score included four studies with 1038 adult patients, 135 of whom (13%) had advanced fibrosis. In the FIB-4 index group, the pooled sensitivity, specificity, and AUROC (area under the ROC curve) at a 1.30 cut-off were 0.844 (0.772–0.901), 0.685 (0.654–0.716), and 0.8496 ± 0.0680, respectively. At a cut-off of 3.25, the sensitivity was lower (0.38), but the specificity was higher (0.96), with an AUROC of 0.8445 ± 0.0981. For the BARD score group, the pooled sensitivity, specificity, and AUROC were 0.74 (0.66–0.81), 0.66 (0.63–0.69), and 0.7625 ± 0.0285, respectively. These results show that the FIB-4 index at the 1.30 cut-off has better diagnostic accuracy than at the 3.25 cut-off, as well as compared to the NFS and BARD scores, although its ability to predict advanced fibrosis in NAFLD remains limited.

The FIB-4 index has also been proposed as a prescreening tool to improve the efficiency of referring patients for specialized liver care, helping prioritize those at higher risk for significant liver disease care [248]. Overall, its diagnostic accuracy surpasses that of other simple non-invasive tests (NITs), including the NFS, AST to platelet ratio index (APRI), and BARD score [234,246,249,250,251,252,253,254] (Table 3).

The negative predictive values (NPV) for all methods (APRI, FIB-4 index, BARD score, and NFS) were above 75% when diagnosing severe fibrosis. The summary specificities of three models (APRI, FIB-4 index, and NFS) exceeded 85% for predicting severe fibrosis, while the BARD score was less accurate compared to the others. Both APRI and FIB-4 index showed summary specificities greater than 95% when detecting severe fibrosis. The specificities of APRI (cutoff of 1.5), FIB-4 index (cutoff of 2.67), BARD score (cutoff of 2), and NFS (cutoff of 0.67–0.676) were 96.1%, 96.5%, 61.3%, and 94.6%, respectively. Only FIB-4 and NFS had summary positive predictive values (PPV) above 70%. [248].

### 4.5. FIB-4 Index and Mortality

MAFLD patients with higher FIB-4 index are associated with increased liver disease and overall mortality [255,256,257,258] (Table 3). When NITs are applied to the general population, NITs did not become better predictor of severe liver disease than expected [259]. In NAFLD with diabetes, FIB-4 index, NFS, and APRI cannot predict liver-related mortality and morbidity [260]. NFS can stratify risk of cardiovascular events and extrahepatic malignancies [261]. FIB-4 index is also associated with all-cause mortality of systemic chronic diseases such as rheumatoid arthritis [262], microscopic polyangiitis, granulomatosis with polyangiitis [263], and chronic obstructive pulmonary disease [264]. The underlying mechanisms of these relationships remain unknown.

FIB-4 Index and Risk of Cardiovascular Disease

The leading cause of mortality in MAFLD patients is CVD, followed by extrahepatic cancer and liver related diseases [265]. MAFLD is an independent risk factor of coronary sclerosis [266], atrial fibrillation (AF) [267], coronary artery disease (CAD), and left ventricular dysfunction [268,269]. In daily clinical practice, we should pay attention to CVD event and control other risk factors, such as hypertension, dyslipidemia, and type 2 diabetes. FIB-4 index appears to be associated with high risk of CVD mortality [258].

### 4.6. Elastosonography

The role of shear wave elastography in both hepatic fibrosis staging and prognostication in different etiologies of liver disease is discussed in a recent guideline [239], highlighting advantages and limitations. The role of hepatic elastosonography in the diagnosis and prognosis in heart failure is present in a recent review [270].

### 4.7. Non-Invasive Methods for Hepatic Steatosis in Mafld

Hepatic steatosis and/or fibrosis are linked to an increased risk of developing new cases of heart failure (iHF), being hospitalized for heart failure (hHF), and higher mortality rates, including cardiovascular death, in both the general population and among heart failure patients [149]. Additionally, changes in hepatic steatosis, as measured by the Fatty Liver Index (FLI), are associated with risks of new heart failure, heart failure-related hospitalizations, and overall mortality, including cardiovascular or liver-related deaths [150]. When analyzing data by age and BMI, and using the non-NAFLD group as a baseline, the risk of incident heart failure tends to increase across groups from regressed NAFLD to incident NAFLD to persistent NAFLD.

According to the latest AASLD guidelines [271], while conventional B-mode ultrasound is widely used, it lacks sufficient sensitivity to detect mild steatosis, especially in individuals with obesity, and offers only a subjective, semi-quantitative assessment of steatosis severity [28,272]. The absence of detectable steatosis on ultrasound does not rule out the presence of metabolic-associated steatosis hepatitis (MASH) or fibrosis. However, ultrasound can be useful for identifying cirrhotic liver morphology or signs of portal hypertension, such as ascites, splenomegaly, or portosystemic collateral vessels. For evaluating hepatic steatosis, the Controlled Attenuation Parameter (CAP), typically used with Vibration-Controlled Transient Elastography (VCTE), provides a semi-quantitative assessment of liver fat but does not accurately quantify or monitor changes in liver fat [273]. MRI with proton density fat fraction (PDFF) is a precise and reliable method for quantifying liver fat, commonly used in clinical research, and its role in clinical practice is growing, especially in tertiary care centers. Although MRI-PDFF is superior to CAP for diagnosing and quantifying liver fat, it is more expensive, less readily accepted by patients, and not a point-of-care technique [273].

Accurate, non-invasive detection and quantification of hepatic steatosis are crucial for managing nonalcoholic fatty liver disease. Available methods include the Fatty Liver Index, MRI, and ultrasound systems like Attenuation Coefficient, Backscatter (BSC)/ultrasound-derived fat fraction, and Speed of Sound (SOS).

1. The fatty liver index (FLI)
2. MAGNETIC RESONANCE IMAGING EVALUATION OF HEPATIC STEATOSIS
3. ULTRASOUND EVALUATION OF HEPATIC STEATOSIS

A. ultrasound-measured attenuation coefficient (AC)
B. Backscatter (BSC)/ultrasound-derived fat fraction
C. Speed of Sound (SoS)

The main characteristics and evidence for liver steatosis evaluation are reviewed in Table 4.

1. FLI

The fatty liver index (FLI) is an algorithm based on waist circumference, body mass index (BMI), triglyceride, and gamma-glutamyl-transferase (GGT), is a noninvasive method of assessing hepatic steatosis and is calculated by the following formula [274]:

*FLI* = (e0.953 × *loge*(*triglycerides*) + 0.139 × *BMI* + 0.718 × *loge*(*GGT*) + 0.053 × *waistcircumference* − 15.745)/(1 + e0.953 × *loge*(*triglycerides*) + 0.139 × *BMI* + 0.718 × *loge*(*GGT*) + 0.053 × *waistcircumference* − 15.745) × 100

A Fatty Liver Index (FLI) score above 60 (FLI > 60) is a recognized marker for metabolic dysfunction-associated steatotic liver disease (MASLD), which is linked to a heightened risk of diabetes and cardiovascular disease. On the other hand, an FLI score below 20 (FLI < 20) rules out the presence of liver fat (steatosis). All individuals with FLI > 60 were diagnosed with MASLD, while none with FLI < 20 showed signs of steatosis, as confirmed by magnetic resonance spectroscopy (MRS). Subjects with FLI > 60 exhibited higher body mass index (BMI) and greater amounts of visceral, subcutaneous, epicardial, extrapericardial, and overall cardiac fat, as well as increased liver fat (assessed by magnetic resonance imaging (MRI) and MRS), insulin resistance (determined using HOMA-IR and OGIS-index), and features of metabolic syndrome. They also had elevated levels of abdominal and cardiac fat (VAT > 1.7 kg, CARD-AT > 0.2 kg), higher blood pressure, and hyperlipidemia, which together indicate a greater risk of developing cardiometabolic diseases [275].

In a Dutch cohort [276] of 4165 patients aged 60–80 years, who had suffered a myocardial infarction (MI) within the past 10 years, the negative association between FLI and cardiovascular disease (CVD) mortality was stronger in women than men, based on the conventional FLI cut-off points. An FLI score greater than 60, signaling the presence of nonalcoholic fatty liver disease (NAFLD), was a predictor of both CVD and overall mortality in post-MI patients, independent of other cardiometabolic risk factors. Additionally, a study conducted in China [277] highlighted a direct and positive relationship between FLI and the prevalence of ischemic heart disease (IHD), suggesting that FLI could enhance the detection of IHD in the general population. Research from Italy [278] on individuals with type 1 diabetes showed that FLI is linked to higher overall mortality and an increased risk of cardiovascular events in this group.

The relationship between changes in FLI and overall mortality during follow-up remained significant for those under 60 years of age, although FLI changes were associated with mortality across all ages, regardless of BMI [150]. FLI is frequently used in clinical and epidemiological studies to distinguish between healthy individuals and those with NAFLD. A systematic review of FLI research [279] revealed that this index is associated with a wide range of biochemical and physiological parameters, including lipid, protein, and carbohydrate metabolism, hormones, vitamins, inflammation markers, and oxidative stress. FLI can serve as a predictor or risk factor for various metabolic and non-metabolic diseases, as well as mortality. It is also used to assess the effects of preventive health interventions, medications, and toxic substances on humans [279]. Additionally, a similar index exists in the United States (US FLI) [280], which is calculated based on factors such as age, race-ethnicity, waist circumference, gamma-glutamyltransferase activity, fasting insulin, and fasting glucose.

2. Magnetic Resonance Imaging Evaluation of Hepatic Steatosis

Accurate diagnosis and monitoring of fatty liver disease are essential for early treatment and prevention of complications. While liver biopsy has traditionally been the gold standard for diagnosis, it is invasive, involves risks, and only samples a small portion of the liver [281]. As a result, non-invasive techniques are becoming more popular for detecting liver fat. Magnetic resonance imaging proton density fat fraction (MRI-PDFF) is currently regarded as the best non-invasive method for measuring liver fat in MASH clinical trials. A 2018 systematic review [282], which included six studies with 635 patients, found that MRI-PDFF had AUROC values of 0.98, 0.91, and 0.90 for differentiating between steatosis grades 0 versus 1–3, 0–1 versus 2–3, and 0–2 versus 3, respectively. The pooled sensitivity and specificity of MRI-PDFF for these same steatosis grade classifications were 0.93 and 0.94, 0.74 and 0.90, and 0.74 and 0.87, respectively. The study also reported positive and negative likelihood ratios for classifying these steatosis grades. A subsequent systematic review in 2021 [283], which analyzed seven studies involving 346 participants (with a median age of 51 years, 59% female, and 46% with diabetes) (studies not present in the previous meta-analysis [282]), demonstrated that a relative reduction of at least 30% in MRI-PDFF is associated with a higher likelihood of histologic improvement and MASH resolution. This supports the use of MRI-PDFF in early-stage MASH clinical trials to non-invasively monitor treatment response and to assist in sample size estimation for studies based on histological evaluations. In 2021, Gidener et al. [284] showed that in patients with NAFLD, liver stiffness measured by magnetic resonance elastography (MRE) is a significant predictor of future cirrhosis. Growing consensus supports that MRI-assessed proton-density fat fraction (PDFF) accurately measures liver fat, while MRE is the most reliable technique for assessing and staging liver fibrosis in NAFLD patients [285]. Additionally, MRE-based liver stiffness measurements are not influenced by the severity of liver fat, unlike ultrasound-based methods [286]. This highlights the expanding role of MRE in clinical practice, aiding in more personalized disease monitoring and patient management. In 2023, the American Association for the Study of Liver Diseases (AASLD) [271] published guidelines recommending the FIB-4 index as an initial screening tool for advanced fibrosis in NAFLD. If the FIB-4 score exceeds 2.67 or if ALT or AST levels remain elevated, further evaluation with MRE is advised to offer a more accurate assessment of fibrosis. Despite its precision and recognition as a reference method for fat quantification, MRI-PDFF remains expensive and has limited availability [282].

3. Ultrasound Evaluation of Hepatic Steatosis

There are three ultrasound (US) techniques currently being used to assess liver fat: attenuation coefficient (AC), backscatter coefficient (BSC), and speed of sound (SoS) [287] (Table 5). The hepatorenal index is calculated as the ratio between US signals reflected by the liver and those reflected by the right kidney’s cortex, which serves as the reference. This method relies on raw US data, excluding any corrections for attenuation in the image. For accurate results, the kidney cortex must be normal. However, this is not a direct or quantitative measure of liver fat. All these techniques use the raw data from the US beam to estimate parameters related to liver fat. The raw data consists of signals that are returned to the transducer, containing a wealth of information such as frequency and signal strength. Each technique utilizes different aspects of this raw data to estimate the amount of liver fat.

A. ultrasound-measured attenuation coefficient (AC)

Several algorithms are currently available for estimating the attenuation coefficient (AC) (Table 5). These include the controlled attenuation parameter (CAP), which measures the slope of the AC over a fixed distance at a single frequency, expressed in decibels per meter (dB/m). Additionally, ultrasound (US) imaging systems offer algorithms that calculate the AC across a range of frequencies within the US beam, reported in decibels per centimeter per megahertz (dB/cm/MHz). Differences between manufacturers’ algorithms may arise depending on the methods used to calculate the AC. Newer AC algorithms are more sensitive than CAP, though they maintain similar specificity [287]. The improved diagnostic accuracy of AC compared to CAP can be attributed to the image-guided approach used for measurement. AC is clinically useful for evaluating hepatic steatosis, with good diagnostic performance overall. However, in patients with higher body mass index (BMI), the increased distance between the skin and the liver capsule could lead to an overestimation of the AC [288].

B. backscatter coefficient (BSC): ultrasound-derived fat fraction (UDFF)

In 2017, Paige et al. [289] evaluated the diagnostic accuracy of two experimental quantitative ultrasound (QUS) parameters—attenuation coefficient and backscatter coefficient—by comparing them to conventional ultrasound (CUS) and MRI-based proton density fat fraction (PDFF) for predicting histology-confirmed liver fat levels in adults with nonalcoholic fatty liver disease (NAFLD). They found that CUS had a grading accuracy of 51.7%. For attenuation coefficient, the raw and cross-validated grading accuracies were 61.7% and 55.0%, respectively. For backscatter coefficient, they were 68.3% and 68.3%, and for MRI-PDFF, they were 76.7% and 71.3%. Interobserver agreement rates were 53.3% for CUS, 90.0% for attenuation coefficient, and 71.7% for backscatter coefficient, with all showing statistically significant differences. In 2020, a study [290] validated the clinical feasibility of a new method called the ultrasound-derived fat fraction (UDFF) tool, which uses an integrated reference phantom for quantitative ultrasound. This tool was assessed for its performance in diagnosing histologic liver steatosis and its correlation with MRI-PDFF. The Pearson correlation coefficient between UDFF and PDFF was 0.87, with 95% limits of agreement at ±8.5%. The tool demonstrated high accuracy in diagnosing steatosis, with area under the receiver operating characteristic curve (AUC) values of 0.97 and 0.95 for MRI-PDFF thresholds of 5% and 10%, respectively. There was no significant correlation between body mass index (BMI) and either UDFF or PDFF. A 2022 study [291] enrolled 56 overweight and obese adolescents and adults who underwent both investigational ultrasound and MRI of the liver in a single visit. The study included multiple UDFF measurements and calculated an overall median. The AUC for UDFF in detecting MRI-PDFF of 5.5% or more was 0.90, showing good diagnostic performance, which did not significantly vary based on the number of UDFF measurements. In 2023, an Italian study [292] with 122 patients showed that UDFF and MRI-PDFF had high agreement and a strong positive correlation. UDFF demonstrated better diagnostic value than conventional ultrasound for detecting steatosis, with AUC values of 0.75 compared to 0.53 for traditional methods. Zalcman et al. [293] found that in a pediatric population, UDFF correlated well with MRI-PDFF, with a mean bias of 0.64%. UDFF had an AUC of 0.95 for detecting steatosis, with a cutoff of 6% yielding 90% sensitivity and 94% specificity. BMI was an independent predictor of UDFF. Ferraioli (2023) [294] showed that attenuation coefficient estimates are influenced by measurement depth, with values decreasing as depth increases. Another study by the same group [295] in 2024 demonstrated that backscatter coefficient decreased by 13.98 dB/cm-steradian for each 1 cm increase in depth, and both skin-to-liver-capsule distance and stiffness were independent predictors of backscatter coefficient. Kubale (2024) [296] reported that UDFF and whole-liver PDFF measurements had a high intra-class correlation coefficient (ICC) of 0.79. Bland-Altman analysis showed a mean difference of 1.5% between UDFF and voxel-based PDFF measurements, with limits of agreement ranging from −11.0% to 14.0%. UDFF showed strong agreement with PDFF, with good diagnostic performance and repeatability, though it exhibited a slight bias towards higher values compared to PDFF. Zhu (2024) [297] found that various ultrasound attenuation-based techniques were well correlated and had good diagnostic performance for hepatic steatosis. However, different techniques had varying threshold values, and combining multiple parameters might enhance diagnostic accuracy for detecting liver fat.

4. Speed of Sound (Sos)

Conventionally, medical US systems assume a Speed of Sound (SoS) for transmitting and receiving beamforming operations. The assumed SoS is typically held constant, usually at 1540 m/s for the entire image. However, because of this assumption, the ultrasound image quality may have a degradation because the different organs may have different SoSs [298]. The SoS is slower in fatty tissue; therefore, as fat increases in the liver, the SoS will decrease.

The SoS measurements for liver tissue can be based on four techniques: focusing, spatial coherence, compounding, and single-path transmission. The first three selected methods were recently considered the most promising categories for SoS measurements [299,300]. A review of the basic science of SoS is available by the AIUM-RSNA QIBA Pulse-Echo Quantitative Ultrasound Initiative [300]. Currently, there is not any published study about factors that can affect the measurement of the BSC or the SoS. However, it should be underscored that the measurement of the BSC is dependent and generally combined with that of the AC. Therefore, the protocol used for this latter must be followed to mitigate the variability in the measurement of liver fat content [287].

## 5. Conclusions

Metabolic syndrome is a complex condition characterized by a combination of factors including abdominal obesity, insulin resistance, high blood pressure, and abnormal lipid levels (high triglycerides and low high-density lipoproteins). This syndrome significantly increases the risk of developing type 2 diabetes mellitus (T2DM) by five to seven times, cardiovascular disease (CVD) by three times, and all-cause mortality by one and a half times.

The definition, the change in nomenclature (from NAFLD/NASH to MAFLD/MASH and now MASLD/MASH), and the pathogenesis are discussed. The relationship between steatosis, MASH, metabolic syndrome and heart failure, diabetes, renal failure, are described. The association between metabolic syndrome and vascular, neurological, pneumological (such as asthma, COPD, OSAS pulmonary hypertension), rheumatological (such as osteoarthritis and rheumatoid arthritis), sexual (impotence and polycystic ovary) and neoplasms are also briefly analyzed.

The primary diagnosis of MASLD involves imaging techniques like ultrasound and MRI to detect fatty liver, along with elevated liver enzyme levels, while ruling out other causes of liver disease. Liver biopsy remains the gold standard for assessing disease severity, but due to its invasive nature, non-invasive methods such as blood tests and elastometry are often used first. The FIB-4 index is commonly used for screening advanced fibrosis, and if necessary, MRI-based methods can provide a more accurate assessment of liver fat and fibrosis, though they are costly and not always readily available. Non-invasive methods to assess liver fat include ultrasound techniques like attenuation coefficient (AC), backscatter coefficient (BSC), and speed of sound (SoS). Recent guidelines suggest using MRI-PDFF as the reference standard for fat quantification, despite its cost and limited availability. Although techniques like CAP have become more common, their specificity is limited in patients with metabolic risk factors. AC, when combined with BSC, shows promising initial results, but standardized protocols are needed to ensure consistency across studies. Overall, while there is still limited data on the prognostic value of these methods, they offer new opportunities for non-invasive evaluation of metabolic and cardiovascular diseases.

This work gives a panoramic view of the metabolic syndrome, from an etiopathogenetic and clinical point of view. Its uniqueness, however, is in the analysis of all the main non-invasive diagnostic systems and in their correlation with clinical and prognostic utility. In fact, it aims to provide the clinical physician (gastroenterologist, internist, diabetologist, pulmonologist, nephrologist, rheumatologist, etc.), the knowledge of this multifaceted and multi-specialist pathology and the tools to be able to study it in a simple and non-invasive way.

## Figures and Tables

**Table 1:** A variety of non-invasive tests (NITs) for identifying severe fibrosis (F3/4) in MASLD.

Index	Formula	Strengths	Weaknesses
**APRI [233]**	AST to platelet ratio index	Simple (only two parameters)	Conflicting results
**NAFLD** **fibrosis score (NFS) [234]**	−1.675 + 0.037 × age (years) + 0.094 × BMI (kg per m2) + 1.13 × impaired glucose tolerance or diabetes mellitus (yes = 1, no = 0) + 0.99 × AST to ALT ratio − 0.013 × platelet (× 109/L) − 0.66 × albumin (g/dL)	Validated globally Accurate	Complex (six parameters) Requires an intermediate group Overpredict in old patients
**BARD [235]**	BMI > 28 kg/m^2^ = 1 point AST/ALT ratio > 0.8 = 2 points Diabetes = 1 point	Very simple	Conflicting results
**FIB-4 SCORE [236,237]**	(age [years] × AST [U/L]/(platelet count [10^9^/L]) × $\sqrt{ALT[U/L]}$)	Simple (only four parameters) Accurate Validated globally	Requires an intermediate group Overpredict in old patients Inferior in patients with T2D?

APRI: AST to platelet ratio index; BARD: BMI, AST/ALT ratio and Diabetes; NFS: NAFLD fibrosis score; FIB-4: fibrosis index based on four factors.

**Table 2:** Methods available for the diagnosis of liver fibrosis in MAFLD.

Methods for Hepatic Fibrosis Diagnosis	Advantages	Disadvantages	Evidences About Hepatic Fibrosis and Cardiovascular Accidents
Hystological Examination			
Biopsy [231,232]	Best standard	Invasive High costs	High
Radiological Examination			
MRI [238]	Not invasive best standard	High costs No diffusion	No data
Serological Examination			
APRI [233]	Not invasive Low costs	High “grey zone”	Good (in more settings)
NFS [234]	Not invasive Low costs	High “grey zone”	Good (in more settings)
BARD score [235]	Not invasive Low costs	High “grey zone” To identify f4	Good (in more settings)
FIB-4 score [236,237]	Not invasive Low costs	High “grey zone” To identify f4	Good (in more settings)
Ultrasonographic examination			
Elastosonography [239]	not invasive low costs	No diffusion Operator-dipendent	Low evidence (few studies)

MRI: Magnetic Resonance Imaging; APRI: AST to platelet ratio index; BARD: BMI, AST/ALT ratio and Diabetes; NFS: NAFLD fibrosis score; FIB-4: fibrosis index based on four factors.

**Table 3:** Summary sensitivities, specificities, PPV, and NPV of APRI, NAFLD Score, BARD score and FIB-4, at various diagnostic thresholds for prediction of severe fibrosis.

APRI					
Cutoff Values	No. of Studies (No. of Patients)	Sensitivity, %, Mean (Range)	Specificity, %, Mean (Range)	PPV, %, Mean (Range)	NPV, %, Mean (Range)
0.452–0.50	5 (579)	72.9 (50.0–87.4)	67.7 (43.1–91.0)	44.8 (22.9–71.0)	89.4 (84.9–95)
0.54–0.98	7 (1351)	68.6 (61.0–76.2)	72.7 (59.4–86.0)	61.4 (46.9–76.2)	77.6 (59.4–94)
1	4 (1101)	43.2 (61.0–76.2)	86.1 (81.0–89.9)	33.5 (26.0–40.0)	89.8 (84.0–95.0)
1.5	4 (682)	32.9 (6.3–70.0)	90.5 (74.5–97.0)	55.5 (40.0–72.1)	79.1 (73.2–87.2)
NFS					
Cutoff Values	No. of Studies (No. of Patients)	Sensitivity, %, Mean (Range)	Specificity, %, Mean (Range)	PPV, %, Mean (Range)	NPV, %, Mean (Range)
(−26.93)–(−2.16)	2 (106)	80.5 (78.0–83.0)	69.5 (69.0–70.0)	NONE	NONE
−1.455	10 (3057)	72.9 (22.7–96.0)	73.8 (42.9–100)	50.4 (24.0–100)	91.8 (81.3–98.1)
(−1.31)–(0.156)	5 (963)	78.2 (69.0–86.4)	71.7 (60.0–83.0)	58.4 (34.0–80.8)	82.1 (54.1–95.0)
0.67–0.676	14 (3896)	43.1 (8.3–100)	88.4 (25.0–100)	66.9 (26.0–100)	88.5 (78.6–100)
0.735	1 (235)	68.4	88.3	53.0	93.5
BARD					
Cutoff Values	No. of Studies (No. of Patients)	Sensitivity, %, Mean (Range)	Specificity, %, Mean (Range)	PPV, %, Mean (Range)	NPV, %, Mean (Range)
1.5	1 (242)	83	59.0	34.0	93.0
2	14 (3057)	75.2 (41.7–100)	61.6 (32.5–88.9)	38.3 (15.0–79.8)	88.7 (49.6–100)
3–4	5 (736)	59.4 (33.3–85.2)	75.1 (59.9–91.8)	55.2 (24.0–69.2)	81.0 (71.4–90.1)
FIB-4 INDEX					
Cutoff Values	No. of Studies (No. of Patients)	Sensitivity, %, Mean (Range)	Specificity, %, Mean (Range)	PPV, %, Mean (Range)	NPV, %, Mean (Range)
1.24–1.45	10 (2759)	77.8 (63.0–90.0)	71.2 (55.5–88.0)	40.3 (24.0–50.6)	92.7 (88.0–98.0)
1.51–2.24	8 (1533)	77.0 (70.6–89.5)	79.2 (67.1–93.6)	66.4 (37.4–85.7)	83.9 (58.6–97.2)
2.67	6 (1910)	31.9 (12.0–63.2)	95.7 (88.3–98.7)	66.0 (51.1–80.0)	85.0 (79.4–92.6)
3.25	6 (1890)	37.3 (5.0–56.0)	95.8 (89.0–100)	72.5 (37.0–100)	87.3 (78.5–94.0)
5.31–10.62	4 (534)	67.5 (50.0–100)	80.4 (54.0–100)	90.0 (80.0–100)	85.1 (80.0–90.2)

APRI: AST to platelet ratio index; BARD: BMI, AST/ALT ratio and Diabetes; NFS: NAFLD fibrosis score; FIB-4: fibrosis index based on four factors.

**Table 4:** Methods available for the diagnosis of liver steatosis in MAFLD.

Methods for Hepatic Steatosis Diagnosis	Advantages	Disadvantages	Evidences About Hepatic Steatosis and Cardiovascular Accidents
**Serological examination**			
Fatty liver index (fli)	Not invasive Low costs	High “grey zone”	Good (in more settings)
**Radiological examination**			
MRI	Not invasive best standard	High costs No diffusion	No data
**Ultrasound examination**			
US-measured attenuation coefficient (AC)	Simple, non-invasive, cheap	Low sensibility	good EVIDENCE
Backscatter (BSC)/US-derived fat fraction (UDFF)	Simple, non-invasive, cheap	Better sensibility	Low EVIDENCE
speed of sound (SoS)	Simple, non-invasive, cheap	Low sensibility	low EVIDENCE

**Table 5:** Algorithms currently commercially available for the estimate of liver fat content with ultrasound systems.

Algorithm	Manufacturer	Name	Unit of Measurement
AC	Canon (Tokyo, Japan)	ATI (attenuation imaging)	dB/cm/MHz
AC	Echosens (Paris, France)	CAP (controlled attenuation parameter)	dB/m
AC	Esaote (Genoa, Italy)	QAI (Q-attenuation imaging)	dB/cm/MHz
AC SoS	E-Scopics (Aix-en-Provence, France) Fujifilm (Tokyo, Japan)	ATT (attenuation) SOS (speed of sound) BSC iATT (attenuation)	dB/m m/s dB/cm-sr dB/cm/MHz
AC	General Electric (Boston, MA, USA)	UGAP (ultrasound derived fat fraction)	dB/cm/MHz
AC	Mindray (Shenzhen, China)	USAT (ultrasound attenuation)	dB/cm/MHz
AC	Philips (Amsterdam, The Netherlands)	ATI (attenuation imaging)	dB/cm/MHz
AC BSC AC + BSC AC + BSC	Samsung (Suwon, Republic of Korea) Siemens (Munich, Germany)	TAI (tissue attenuation imaging) TSI (tissue scattering imaging) USFF (ultrasound fat fraction) UDFF (ultrasound derived fat fraction)	dB/cm/MHz - % %
AC SoS	SuperSonic Imagine (Aix-en-Provence, France)	ATT PLUS (plane-wave ultrasound attenuation) Ssp PLUS (plane-wave ultrasound speed of sound)	dB/cm/MHz m/s

AC, attenuation coefficient; BSC, backscatter coefficient; dB/cm/MHz, decibel/centimeter/megahertz; dB/m, decibel/meter; m/s, meter/second; sr, steradian; SoS, speed of sound.

## Data Availability

Not applicable.
